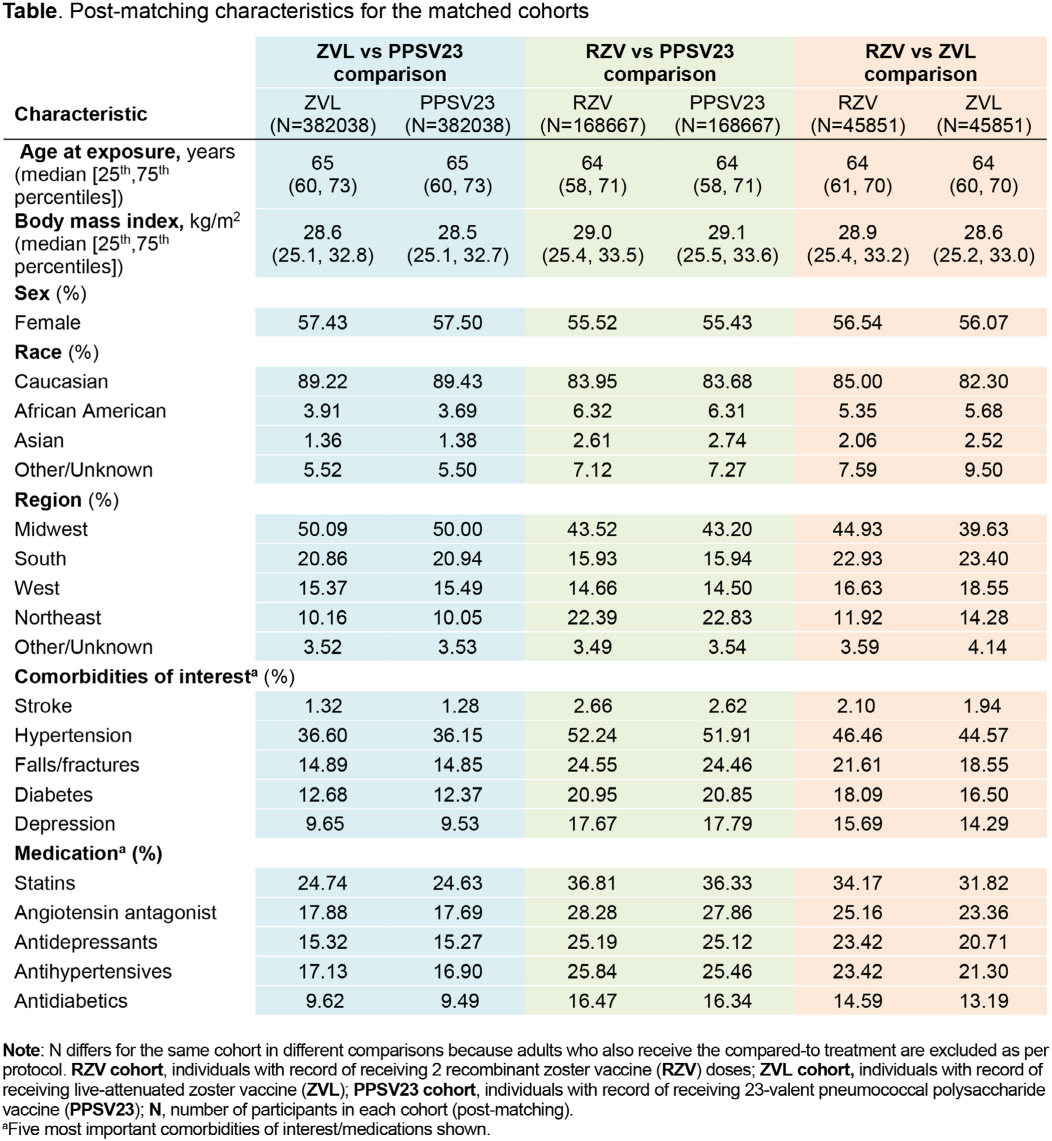# Recombinant zoster vaccine and reduced risk of dementia: matched‐cohort study using large‐scale electronic health records and machine learning methodology

**DOI:** 10.1002/alz.088064

**Published:** 2025-01-09

**Authors:** Patrick Schwab, Robyn Widenmaier, Halima Tahrat, Maria Littmann, Bruno Anspach, Carolyn Buser‐Doepner, Andreas Georgiou, Max Horn, Sanjay Kumar, Vitaly Polisky, Aleksei Triastcyn, Cornelia M Van Duijn, Pascal Geldsetzer

**Affiliations:** ^1^ GSK, Baar, Zug Switzerland; ^2^ GSK, Toronto, ON Canada; ^3^ GSK, Rockville, MD USA; ^4^ GSK, Heidelberg, Baden‐Württemberg Germany; ^5^ GSK, Collegeville, PA USA; ^6^ University of Oxford, Oxford United Kingdom; ^7^ Stanford University, Stanford, CA USA

## Abstract

**Background:**

With the world population aging, the number of individuals living with dementia is expected to increase significantly. Vaccination against herpes zoster (HZ) with the live‐attenuated zoster vaccine (ZVL) was associated with a lower risk of being diagnosed with dementia in previous studies. We aimed to determine whether the recombinant zoster vaccine (RZV) immunization is also associated with a reduced risk of dementia diagnosis.

**Methods:**

This retrospective, observational, matched‐cohort study of adults aged ≥50 years used data from the United States Optum® de‐identified Electronic Health Record data set, covering the period 01‐Oct‐2007–30‐Sep‐2023. A comparison cohort comprising adults vaccinated with the pneumococcal polysaccharide vaccine (PPSV23) was created to mitigate selection bias. Matched cohorts were then formed to compare those vaccinated with ZVL vs PPSV23, RZV vs PPSV23, and RZV vs ZVL, with neither comparison arm having been exposed to the comparator vaccine. Matching (1:1) used a propensity score generated with non‐linear estimators based on 394 variables indicating past diagnoses, medication use, preventive health service uptake, and healthcare service utilization. Using the Nelson‐Aalen estimator, the relative risk (RR) for dementia diagnosis (using ICD‐9/‐10 codes) was compared between the three matched cohorts, at 3 and 5 years post‐vaccination.

**Results:**

Post‐matching characteristics were balanced between cohorts (**Table**). Compared to PPSV23, ZVL significantly reduced 3‐year (RR: 0.86, 95% confidence interval [CI]: 0.86‐0.90; *p<0.0001*) and 5‐year (RR: 0. 92, 95%CI: 0.89‐0.95; *p<0.0001*) dementia risk. RZV significantly reduced 3‐year (RR: 0.76, 95%CI: 0.69‐0.84; *p<0.0001*) and 5‐year (RR: 0.80, 95%CI: 0.71‐0.90; *p<0.0005*) dementia risk, when compared to PPSV23. Compared to ZVL, RZV was also associated with a significant reduction of 3‐year (RR: 0.73, 95%CI: 0. 60‐0.89; *p<0.005*) and 5‐year (RR: 0.77, 95%CI: 0.64‐0.92; *p<0.005*) dementia risk.

**Conclusion:**

HZ immunization was associated with a reduced risk of dementia at 3 and 5 years post‐vaccination compared to PPSV23 immunization. RZV was associated with a reduced risk of dementia compared to ZVL at 3 and 5 years post‐vaccination.